# Special Offer for Students

**Published:** 1889-02

**Authors:** 


### SPECIAL OFFER FOR STUDENTS.

It is intended to make the journal the special medium for students. In its advertising columns will be found the largest variety of goods manufactured in this country. Its pages do not advertise the product of one house only, but give place to the leading specialties manufactured by the leading houses, thus affording an opportunity of making a selection. Every manufacturer has some leading article that he makes better than any other house, because of special advantages for manufacturing, etc.; and by consulting our advertising pages the student can find out where to purchase such articles to the best advantage.

The same is true regarding bargains. Every now and then certain houses desire to hold out inducements to students to purchase goods from them, and consequently offer some special line of goods at very low prices. The student surely cannot afford to let such opportunities pass, and the place to find such bargains is in our advertising columns.

Those students who desire to subscribe for the INTERNATIONAL can do so by sending two dollars for the remainder of the year. Those who desire Stowell's Atlas can have it also by enclosing $3.50. If it is desired to have it sent by mail, twenty-five cents additional must be included; otherwise it will be sent by express, at the expense of the subscriber.

Subscribe for the INTERNATIONAL, the organ of the profession, and you will not regret it.

